# Dynamic designing of microstructures by chemical gradient-mediated growth

**DOI:** 10.1038/ncomms7584

**Published:** 2015-03-13

**Authors:** Tae Soup Shim, Seung-Man Yang, Shin-Hyun Kim

**Affiliations:** 1 Department of Chemical and Biomolecular Engineering, KAIST, Daejeon 305-701, Republic of Korea

## Abstract

Shape is one of the most important determinants of the properties of microstructures. Despite of a recent progress on microfabrication techniques, production of three-dimensional micro-objects are yet to be fully achieved. Nature uses reaction–diffusion process during bottom-up self-assembly to create functional shapes and patterns with high complexity. Here we report a method to produce polymeric microstructures by using a dynamic reaction–diffusion process during top-down photolithography, providing unprecedented control over shape and composition. In radical polymerization, oxygen inhibits reaction, and therefore diffusion of oxygen significantly alters spatial distribution of growth rate. Therefore, growth pathways of the microstructures can be controlled by engineering a concentration gradient of oxygen. Moreover, stepwise control of chemical gradients enables the creation of highly complex microstructures. The ease of use and high controllability of this technology provide new opportunities for microfabrication and for fundamental studies on the relationships between shape and function for the materials.

## Abstract (web summary)

While microparticle shape can have significant influence on their properties, simple ways to control shape are difficult to achieve. Here, the authors report a method giving a high degree of control over the shape of such structures by generating gradients of oxygen concentration during photopolymerization.

## Graphical abstract

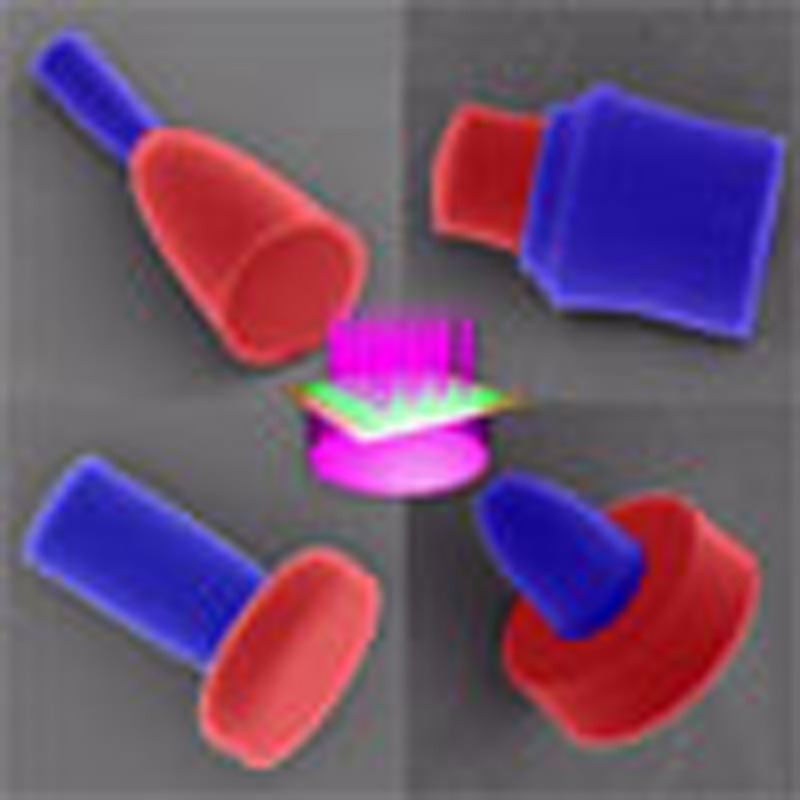

Biological species have evolved in response to environmental factors such as temperature, humidity, and chemical and biological stimuli to form microstructures with shapes that provide the most advantageous function^1^. This process sometimes leads to periodic and hierarchical natural structures with unique optical^2^, mechanical^3^ or biological^4^ functions, which are crucial for the survival and proliferation of the species. Chemical gradients are one of key factors that determine the characteristics of natural structures. For example, microorganisms form fractal patterns in nutrient-poor environments owing to their chemotactic responses^5^. Stripe patterns in animals such as the marine angelfish *Pomacanthus* are believed to be formed by reaction–diffusion processes of morphogens as predicted by Turing^6^. Since these patterns and structures are created dynamically in response to the environment, they are mostly complex and diverse, and thus cannot be easily prepared using static conditions. Engineering chemical gradients into dynamic system should thus be highly promising for structural and compositional design of materials, but such approaches have yet to be fully developed because it is much more difficult to control dynamic conditions than static conditions. Recently, the shape and composition of artificial inorganic microstructures have been shown to be dynamically controlled by applying an external flux of chemicals, which resembles the growth of natural structures^7^. However, the lack of a uniform environment throughout the reaction sites in the reported study yields a field of non-uniformly shaped microstructures. Production of microstructures with uniform 3D features still remains an important challenge.

We address the problem of non-uniformity and are able to create 3D identical copies of microstructures by using a strategy that uses the internal flux of chemicals. A chemical gradient is spontaneously created at each reaction site during photopolymerization, and this gradient changes during the reaction time following a reaction–diffusion process. Therefore, the gradient profile is determined by the ratio of the rate of diffusion to the rate of reaction, as well as by boundary conditions. We generate three distinct growth pathways of polymeric microstructures by controlling the gradient profile. Freezing the polymeric microstructures being formed during one of these pathways when they have the same environments in all reaction sites results in all copies of these structures forming the same 3D shape. Moreover, the microstructures can be further designed by stepwise selection of these pathways, thereby providing higher levels of complexity. Therefore, this new technology overcomes limitations of conventional static fabrication methods such as micromoulding with hard templates^8^ or emulsion drops^9^ and conventional photolithography, which only produce microstructures with pre-determined shapes unless numbers of fabrication steps or complex system design are conducted^10^. More importantly, facile control of the dynamic conditions through selection of the pathway and freezing the reaction provides high reproducibility as well as unprecedented control over the shape and composition of the microstructures.

## Results

### Distinctive growth pathways of polymeric microstructures

**Figure 1:**
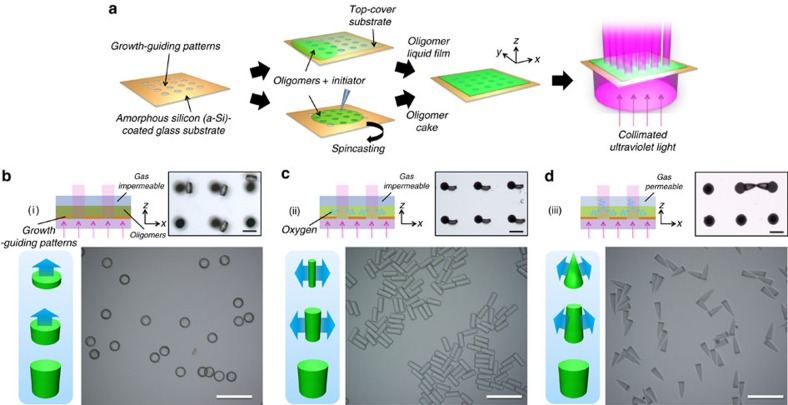
Growth of polymeric microstructures in three different pathways. (**a**) Schematic illustration of photopolymerization with growth-guiding patterns. The patterns are prepared by selective etching of a-Si film coated on the glass substrate. Monomers are coated on the pattern by either infiltration from an additional cover slip or by spin casting, and are then irradiated by collimated ultraviolet light through the growth-guiding patterns to effect photopolymerization. (**b**–**d**) Schematic diagram showing the photopolymerization condition (top left), optical microscope image of polymerized microparticles on the growth-guiding pattern (top right), schematic diagram showing the direction of the growth of the microstructures (bottom left) and optical microscope images of monodisperse microparticles made by the growth pathway (bottom right). Bottom-to-top growth occurs in the absence of oxygen diffusion (**b**), centre-to-side growth occurs when there is diffusion of dissolved oxygen (**c**) and bottom-centre-to-top-edge growth occurs when there is an additional source of oxygen at the top (**d**). Scale bars, 100 μm.

In photopolymerization, the lifetime of the radicals is highly sensitive to oxygen concentration; oxygen molecules rapidly consume radicals to form chain-terminated peroxide molecules. This interruption of the propagation is called oxygen inhibition and is usually undesired with a few exception^11^; for example, Li group used oxygen inhibition for micropatterning^12^. We use oxygen inhibition to spatially control the polymerization rate during the photolithography process. Localized ultraviolet irradiation generates radicals, which are then rapidly consumed by oxygen molecules dissolved in the monomer. This lowers the local oxygen concentration, thereby leading to diffusion of oxygen from the surroundings. The diffusion-induced concentration gradient of oxygen can lead to a spatial distribution of the propagation rate in each reaction site. We study this reaction–diffusion process by selectively irradiating a monomer film containing a photoinitiator through a growth-guiding pattern as shown in Fig. 1a; the polymerized microstructures grow over the ultraviolet irradiation time.

The influence of diffusion becomes significant when the timescale of diffusion is comparable to that of oxygen depletion caused by the termination reaction. The diffusion timescale, *τ*_diff_, of oxygen across the transparent window in the growth-guiding pattern can be estimated as *R*^2^*D*_O_^−1^, where *R* is the radius of the window and *D*_O_ is the diffusion coefficient of oxygen. The rate of oxygen depletion can be approximated to be the same as that of radical formation because the oxygen-induced termination is much faster than radical formation, and inter-radical termination is negligible at the initial stage of polymerization. The rate of radical formation is expressed as *ϕε*[PI]*I*_0_, where *ϕ* is the quantum yield of radical production, *ε* is molar extinction coefficient of the photoinitiator, [PI] is molar concentration of photoinitiator and *I*_0_ is the intensity of the ultraviolet light. Therefore, the oxygen depletion timescale, *τ*_depl_, can be estimated as [O_2_]_0_(*ϕε*[PI]*I*_0_)^−1^, where [O_2_]_0_ is the molar concentration of oxygen initially dissolved in the monomer. The Damköhler number, Da, can be defined as the ratio of the diffusion to depletion timescales:


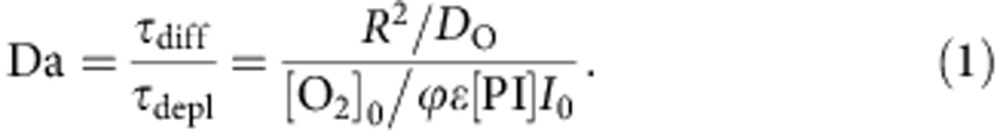


The value of Da indicates the importance of diffusion in polymerization. When Da>>1, diffusion is relatively slow and insignificantly influences the reaction. For example, flow lithography, developed by the Doyle group, is designed to have a low value of *τ*_depl_ to achieve fast polymerization, thereby leading to negligible influence of diffusion on polymerization; microparticles with the same dimensions and shape as the photomask are repeatedly generated in a microfluidic device, where the oxygen inhibition prevents polymerization of the monomer only on the surface of the oxygen-permeable microchannel, thereby ensuring a continuous flow^13^. When Da≪1, diffusion is relatively fast so that oxygen concentration is invariant along the whole reaction site during ultraviolet irradiation until the oxygen in the entire system is almost depleted. By contrast, when Da~1, a concentration gradient of oxygen is generated in the reaction site. This gradient leads to a spatial distribution of the polymerization rate, thereby enabling the creation of polymeric microstructures with shapes that are inaccessible when using other techniques. We used three different sets of experimental systems to investigate the influence of oxygen diffusion on the growth of the microstructures: (i) Da>>1, (ii) Da~1 with only the oxygen that was initially dissolved and (iii) Da~1 with an additional oxygen source.

### Pathway 1 Bottom-to-top growth

In the case of Da>>1, the growth is barely influenced by the oxygen diffusion and the polymeric structures grow along the light propagation direction as shown in Fig. 1b. As ultraviolet light propagates through the reaction site, the photoinitiator absorbs the light, thereby leading to a gradual decrease of ultraviolet intensity along the propagation direction; this decrease can be calculated using the Beer–Lambert law:


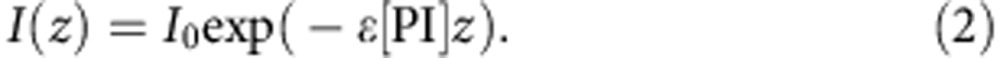


This vertical intensity profile leads to a bottom-to-top growth of the polymerized structure, while maintaining its cross-sectional shape. For example, the SU-8 photoresist, which has a value of *D*_O_ as small as O(10^−14^) m^2^ s^−1^, exhibits such a growth behaviour when ultraviolet light is irradiated through a growth-guiding pattern containing an array of circular windows each with an *R* of 15 μm; in this case, Da~O(10^3^). Therefore, the height of the resultant microstructures can be controlled by ultraviolet irradiation time as shown in top right panel of Fig. 1b.

### Pathway 2 Centre-to-side growth

In the case of Da~1, oxygen diffusion dominates growth pathway. Oxygen-induced radical termination leads to an abrupt decrease of the oxygen concentration at the reaction site, whereas the oxygen concentration initially remains unchanged at the idle non-ultraviolet-irradiated sites. Oxygen then diffuses from the idle sites to the reaction sites, thereby creating a concentration gradient of oxygen and a spatial distribution of the propagation rate. To study this, we used poly(ethylene glycol) diacrylate (PEGDA), which has a value of *D*_O_^PEGDA^=2.84 × 10^−11^ m^2^ s^−1^ and Da=4.74 for the same growth-guiding pattern used for SU-8. To exclude influx of oxygen from the outside, a liquid film of PEGDA was covered with a gas-impermeable glass slide; only oxygen dissolved in PEGDA participates in the reaction–diffusion process. The polymerized structure grows from the central axis of cylindrical reaction site, in which the concentration of oxygen is lowest, to the side during ultraviolet irradiation, while maintaining its height during the growth, as shown in Fig. 1c.

### Pathway 3 Bottom-centre-to-top-edge growth

Including a source of oxygen at the top of the PEGDA monomer film can alter the concentration profile of oxygen and therefore the growth of the microstructures. To carry out such an experiment, a glass slide coated with a thin layer of oxygen-containing poly(dimethylsiloxane) (PDMS) was used to cover the film. Vertical oxygen flux creates a gradual decrease of oxygen concentration from the top. Therefore, the combination of lateral and vertical oxygen flux make the microstructure grow from the centre of the bottom surface of reaction site in which the oxygen concentration is lowest to form cone shape, and then transforms to a full cylinder over time. This bottom-centre-to-top-edge growth is shown in Fig. 1d. It is noteworthy that 3D microstructures can be synthesized from a 2D growth-guiding pattern with unidirectional ultraviolet light.

### Quantitative analysis of growth behaviours

**Figure 2:**
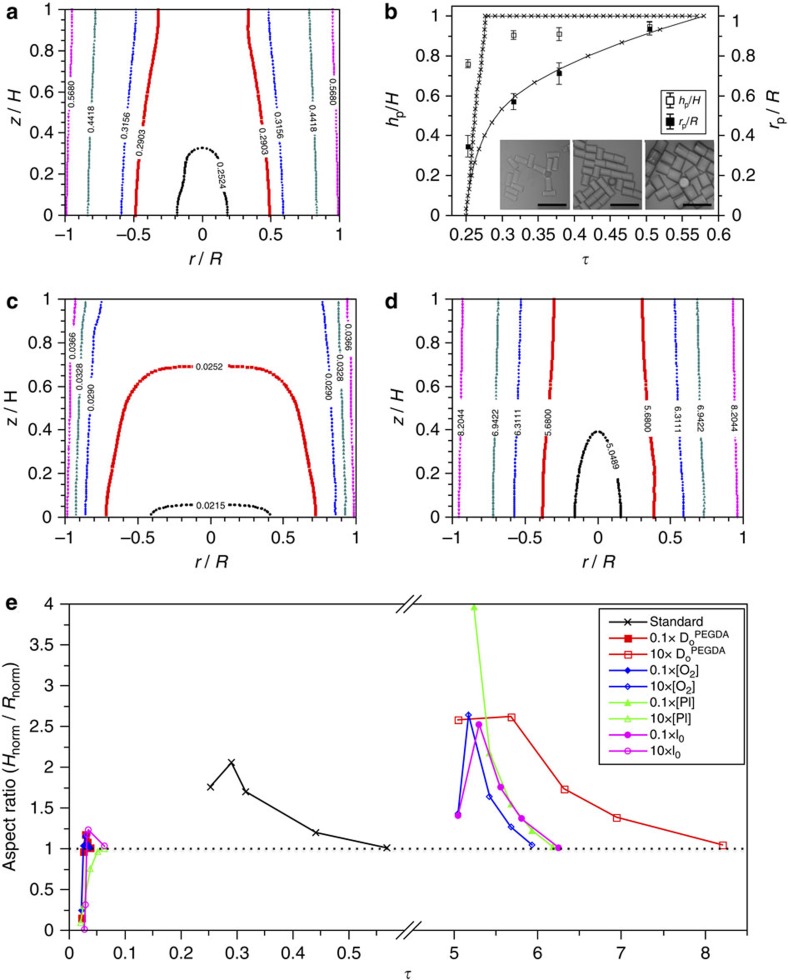
Analysis of growth behaviours. (**a**) Time-dependent contours at which the fraction of monomers consumed by propagation of the reaction, *φ*_p_, is 0.1 at the reaction site, where only dissolved oxygen participates in the reaction–diffusion process. The abscissa and ordinate are normalized width, *r*, and height, *z*, by the radius of the transparent window, *R*, and the height of the monomer film, *H*, respectively. Ultraviolet irradiation time, *t*, is non-dimensionalized by the diffusion timescale of *R*^2^*D*_O_^−1^ to be *τ*=*tD*_O_*R*^−2^, where *D*_O_ is the diffusion coefficient of oxygen. For PEGDA, *D*_O_^PEGDA^ is 2.84 × 10^−11^  m^2^ s^−1^. The Damköhler number, Da, is 4.74. (**b**) Time dependence of the normalized height, *h*_p_/H, (empty squares, left ordinate) and bottom radius, *r*_p_/R, (filled squares, right ordinate) of microcylinders prepared by centre-to-side growth. The three optical microscope (OM) images in the inset show microcylinders made by *τ* values of 0.25 (left), 0.325 (middle) and 0.5 (right), respectively. The calculated values of *h*_p_/H and *r*_p_/R for *φ*_p_=0.1 are shown with solid lines. The error bars indicate s.d.; scale bars, 100 μm. (**c**,**d**) Time-dependent contours for *φ*_p_=0.1 where *D*_O_ is (**c**) 0.1 × *D*_O_^PEGDA^ (Da=47.4) and (**d**) 10 × *D*_O_^PEGDA^ (Da=0.474). (**e**) Time-dependent change of aspect ratio of contours for *φ*_p_=0.1, (*h*_p_/H)/(*r*_p_/R), where *D*_O_ (red), [O_2_] (blue), [PI] (green) and [*I*_0_] (purple) are adjusted by a factor of 10 or 0.1 from **a** to investigate the importance of Da.

The diffusion-induced growth pathways can be quantitatively analysed by Fick’s second law of diffusion containing the reaction term:^14^


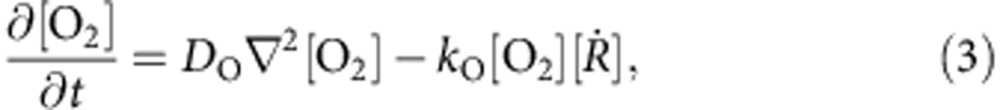


where *k*_O_ is the rate constant for the oxygen-induced termination reaction and 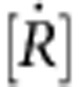 is the molar concentration of radicals determined by rates of photolysis and termination. The solution of equation (3) predicts time-dependent concentration profiles of oxygen and radical (see Supplementary Discussion and Supplementary Fig. 1 in the Supplementary Information for details of the calculation). The propagation rate is proportional to product of 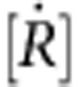 and monomer concentration [*M*]:


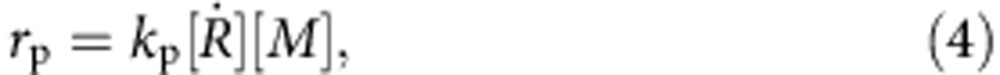


where *k*_p_ is the rate constant for the propagation reaction. Microstructures made by radical polymerization take shape when the fraction of monomer consumed by propagation of the reaction, *φ*_p_, reaches the range of 0.02–0.1 (refs 14, 15). Time-resolved distribution of *φ*_p_ and contours for *φ*_p_=0.1 in the absence of an additional source of oxygen are shown in Supplementary Movie 1 and Fig. 2a, respectively. The contour moves from the centre to the side over time. The growth is quantitatively comparable with experimental observation as shown in Fig. 2b. The height and diameter of the microcylinders prepared by four different ultraviolet irradiation times with same growth-guiding pattern are in good accord with the change of the contour at *φ*_p_=0.1. There is no propagation of the structure until *τ*~ Da^−1^, owing to the fast consumption of radicals by oxygen, and then the reaction suddenly proceeds as soon as oxygen is almost depleted; this is confirmed by the calculation shown in Supplementary Fig. 2a. The height of the contour abruptly increases to be the thickness of the monomer film, whereas the central diameter of the cylinder slowly increases over time. Therefore, we can control the diameters of uniform microcylinders by adjusting ultraviolet irradiation time as shown in the insets of Fig. 2b, where the heights of the microcylinders remain unchanged. The contours for *φ*_p_=0.2 and 0.3 are additionally plotted in Supplementary Fig. 2b, which show larger deviations from the experimental observation in comparison with that of *φ*_p_=0.1.

To study the influence of the diffusion rate on the growth, we calculated the time-dependent contours with one order of magnitude lower and higher values of *D*_O_. For a value of 0.1 × *D*_O_^PEGDA^, the structure promptly forms a plateau and grows upward as shown in Fig. 2c; this is consistent with bottom-to-top growth of the SU-8 structure. The early and fast growth of the structure is attributed to a lack of oxygen supply from the idle sites due to slow diffusion. By contrast, a value of 10 × *D*_O_^PEGDA^ provides centre-to-side growth as shown in Fig. 2d. Although this trend is similar to the growth with a value of 1 × *D*_O_^PEGDA^, there is a long latent period before the onset of the growth, and the growth becomes relatively fast; this combination of a long latent period and fast growth make the control of the aspect ratio of the polymerized structure difficult. Higher values of 100 × *D*_O_^PEGDA^ and 1,000 × *D*_O_^PEGDA^ also result in a growth behaviour similar to that of 10 × *D*_O_^PEGDA^, as shown in Supplementary Fig. 3. Plotting the time-dependent change of the aspect ratio of the resultant structure, to quantitatively compare the growths as shown in Fig. 2e, shows that all the structures eventually display an aspect ratio of 1, if given enough time to grow regardless of Da, as we expect from conventional photolithography. When Da~O(10), which includes 0.1 × *D*_O_^PEGDA^, 0.1 × [O_2_], 10 × [PI] and 10 × *I*_0_, structures rapidly grow from the bottom to the top with a short delay, thereby leading to steep increase of the aspect ratio from 0 to 1; diffusion is still effective at the boundaries between reaction and idle sites, which makes aspect ratio slightly larger than 1 before the value becomes 1. By contrast, when Da~O(10^−1^), which includes 10 × *D*_O_^PEGDA^, 10 × [O_2_], 0.1 × [PI] and 0.1 × *I*_0_, structures slowly grow from the centre to the side with a long delay, thereby leading to gentle decrease of the aspect ratio to 1; the aspect ratio can initially increase until the structure reaches the top surface. The time-dependent growth of contour with *φ*_p_=0.1 for each case is shown in Supplementary Fig. 4. These results imply that a single parameter, that is, Da, governs the overall growth pathway.

### Fabrication of polymeric microstructures by single step

**Figure 3:**
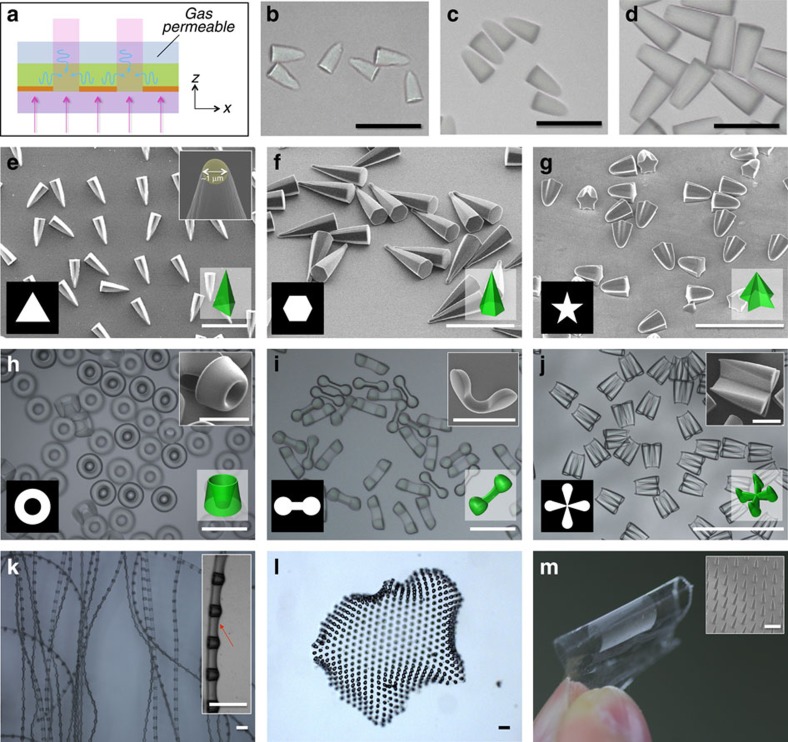
Microstructures created by bottom-centre-to-top-edge growth. (**a**) Schematic of photopolymerization system with additional oxygen supply from the top (same as Fig. 1d). (**b**–**d**) Optical microscope images of microparticles prepared by bottom-centre-to-top-edge growth at *τ*=1.063 (**b**), 1.125 (**c**) and 1.25 (**d**). (**e**–**g**) Scanning electron microscope images of pyramid-type microparticles made by using growth-guiding patterns with (**e**) triangular, (**f**) hexagonal and (**g**) star-shaped windows. Inset in **e** shows that the radius of the apex is as small as 1 μm. (**h**–**j**) Optical microscope images of microparticles with round- or cone-shaped top surface made by (**h**) annular, (**i**) bi-lobed and (**j**) four-lobed windows. Inset images in **h**–**j** are magnified scanning electron microscope images of the corresponding microstructures. For the images of **e**–**j**, window shapes and illustrated resultant structures are included. (**k**–**m**) Optical microscope images of strings composed of a series of domes connected with a thin film (**k**), optical microscope image of a microcone array whose interstices is filled with a thermo-responsive hydrogel of poly(N-isopropylacrylamide) (**l**) and image of a microcone array formed on the surface of the polymeric film (**m**). Insets of **k**,**m** show details of the microstructures, where the arrow in **k** indicates the concave thin film. Scale bars, 100 μm (**b**–**l** and inset images of **k** and **m**). Scale bars, 50 μm (for insets of **h**–**j**).

As described above, the growth pathway can be further modified by introducing an additional oxygen source. This oxygen source influences the boundary condition for diffusion, while the value of Da remains unchanged as O(1). For example, when the monomer film is covered by an oxygen-containing PDMS layer, as depicted in Fig. 3a, a concomitant flux of oxygen into the film leads to a different growth pathway. This growth is studied by adjusting the ultraviolet irradiation time, as shown in Fig. 3b–d. The microstructure takes initially a shape of cone, which then grows to top-cut cone and eventually to full cylinders, so denoted as bottom-centre-to-top-edge growth. By using this type of growth, unusual spired microstructures can be prepared. For example, we can with high fidelity create pyramid-shaped microparticles whose bottom surfaces have triangular, hexagonal and star shapes by using such designs in growth-guiding patterns as shown in Fig. 3e–g, respectively. The radius of the apex can be reduced to be as small as 1 μm as shown in the inset of Fig. 3e. The resultant structures are highly monodisperse and uniform, as shown in the low-magnification images of Supplementary Fig. 5, which proves that the chemical gradients are uniformly controlled over a large area. The design of the pattern can be further diversified. Annular windows allow the creation of volcano-shaped microparticles as shown in Fig. 3h. Bi-lobed windows yield special microparticles each of which has two highest domes connected by a concave thin film, as shown in Fig. 3i. When using a relatively thick monomer solution film, four-lobed windows produce microstructures composed of four sharp cones connected by four films, forming a cross shape, as shown in Fig. 3j. In a similar manner, a series of domes can be connected with relatively thin interstitial films to form a flexible string, as shown in Fig. 3k. In addition, an array of microstructures can be produced by infiltrating monomer into the interstitial space between the microstructures as shown in Fig. 3l or by creating a thin polymer film before growing the microstructures as shown in Fig. 3m.

### Stepwise fabrication of hybrid microstructures

**Figure 4:**
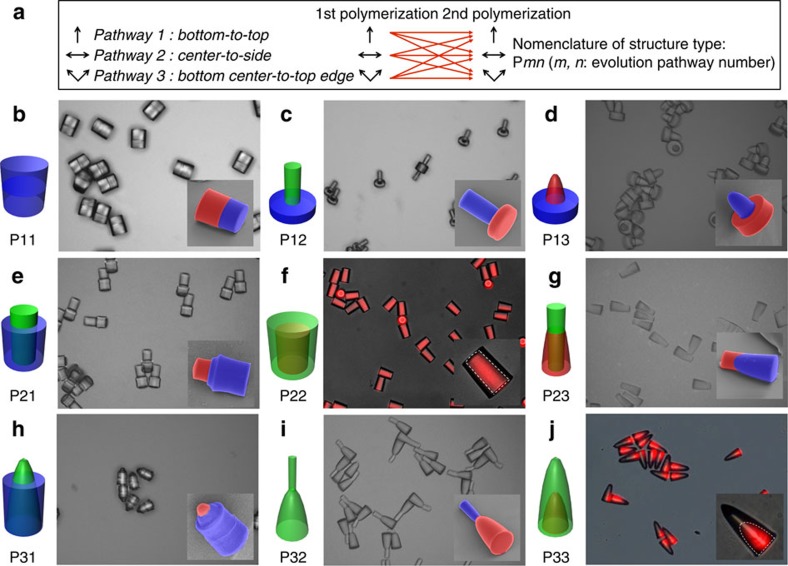
Stepwise design of polymeric microstructures with controlled shape and composition. (**a**) Description of stepwise polymerization to create microstructures with advanced complexity. One of the three different growth pathways (that is, bottom-to-top (tagged with 1), centre-to-side (tagged with 2) and bottom-centre-to-top-edge (tagged with 3)) is independently selected in each of the two polymerization steps, thereby providing nine distinctive microstructures from a single growth-guiding pattern. (**b**–**j**) Sets of schematic illustrations, optical microscope (or fluorescence microscope) images and scanning electron microscope images of nine distinctive microparticles, where the parts made by the first and second steps are coloured red and blue in all SEM images, respectively. For the fabrication of these microparticles, three different monomers are used: SU-8, ethoxylated trimethylolpropane triacrylate (ETPTA) and PEGDA, which in the schematics are shown in blue, red and green, respectively. Images of microparticles with engulfed structures, that is, (**f**) P22 and (**j**) P33, are taken using a fluorescence microscope after selectively staining the interior of the structure with the fluorescent dye rhodamine 6G; this staining delineates the boundary (denoted with the dotted lines in the insets) between the interior and surface parts of the particle.

Additional shapes and compositions can be designed for the microstructures by stepwise application of any two of the three different growth pathways. This combination provides nine (that is, 3 × 3) distinctive types of microstructures even with a single growth-guiding pattern, as described in Fig. 4a. To demonstrate this, we used a growth-guiding pattern containing an array of circular windows and created nine different types of microparticles as shown in Fig. 4b–j. All types are labelled as ‘P*mn*’, where *m* and *n* indicate the growth pathway used for the first and second steps of polymerization, respectively. The microparticles are precisely designed to have unique internal and external structures by controlling the oxygen profile and ultraviolet dose. In addition, the two parts of each microparticle can be made from either the same or different monomers to provide compositional complexity as shown in Fig. 4b–j. This approach is potentially important to functionalize the microparticles. For example, the microparticles tagged with ‘P13’ are designed to have a hydrophobic disk made from SU-8 and a hydrophilic rod made from PEGDA, thereby rendering them amphiphilic; these microparticles can serve as interfacial stabilizers as shown in Supplementary Fig. 6.

## Discussion

The design and production of microparticles and micropatterns with precisely determined shapes and compositions are very important in all fields of material science and engineering. The reaction–diffusion process we have described here enables precise control over the shape and composition of microstructures, which will provide opportunities for microfabrication of new polymeric structures with new applications. For example, pyramid- and cone-shaped microparticles are useful as remotely controllable microneedles that may be used to inject a therapeutic agent onto a target surface; their rotational and translational motions can be independently controlled with an external magnetic field by endowing these microneedles with a permanent magnetic moment, as shown in Supplementary Fig. 7 and Supplementary Movie 2. In addition, our technique can be used to easily prepare microarrays of inverted P12-type microstructures (shown in Supplementary Fig. 8), whose sharp edges can serve as a pinning line of the interface between two immiscible fluids, thereby facilitating the preparation of superhydrophobic or superoleophobic surfaces. Use of a stimulus-responsive hydrogel, formed in the interstices between cone arrays, enables the folding of the hydrogel film with a desired degree of curvature as shown in Supplementary Fig. 9, where this degree of curvature is mainly determined by a slope of each cone; this is important for stimulus-triggered encapsulation and release. Microparticles that are stepwise designed to have two domains with different shapes and compositions are used as advanced building blocks to study self-assembly and interfacial stabilizers in order to immobilize emulsions or aerosol interfaces^16^,^17^,^18^,^19^,^20^. We therefore expect this reaction–diffusion process, which can precisely control the shape and composition of polymeric microstructures, to benefit the wide range of applications (beyond the above examples) that would require the use of specialized types of microparticles and patterns.

## Methods

### Preparation of growth-guiding pattern

An amorphous silicon (a-Si) layer, with a thickness of 200 nm, was coated on a sodalime glass wafer using plasma-enhanced chemical vapour deposition. A positive photoresist (AZ 9260, AZ Electronic Materials) was micropatterned on the substrate by conventional photolithography, which then served as an etching mask of a-Si film for reactive ion etching with SF_6_ (VSRIE-400A, Vacuum Science). The photoresist pattern was finally dissolved out by acetone, yielding a growth-guiding pattern. The array of growth-guiding patterns was typically made with the size of 1 × 1 cm^2^ on the 3 × 3 cm^2^ a-Si-coated glass substrate to produce ~10^4^ microparticles in single ultraviolet exposure.

### Fabrication of microstructures

For the bottom-to-top growth, the epoxy-based photopolymer SU-8 10 (Microchem) was spin coated to be 30 μm thick on the growth-guiding pattern and baked for complete evaporation of residual solvents. Ultraviolet light with an intensity of 14 mW cm^−2^ s^−1^ was then illuminated onto the SU-8 film through the growth-guiding patterns. Subsequently, the SU-8 was baked at 95 °C and the unpolymerized parts were washed out with propylene glycol monomethyl ether acetate (Sigma-Aldrich). For the centre-to-side growth, either PEGDA (Mn 575 g mol^−1^, Sigma-Aldrich) or ETPTA (Mn 428 g mol^−1^, Sigma-Aldrich) containing 5% w/w of Irgacure 2100 (BASF) as a photoinitiator was infiltrated into a gap between the growth-guiding pattern and slide glass, where the gap thickness was controlled by spacers of PDMS film; the thickness was varied from 15 to 110 μm. After ultraviolet irradiation through the growth-guiding patterns, unreacted prepolymers were washed out with ethanol. For the bottom-centre-to-top-edge growth, a 10-μm-thick PDMS film was coated on the glass slide and then the procedures used for the centre-to-side growth were followed.

## Author contributions

T.S.S designed the research, and S.-M.Y. and S.-H.K. supervised the research. T.S.S. carried out all experiments and computer calculations. T.S.S. and S.-H.K. discussed and interpreted the results. T.S.S. and S.-H.K. prepared the manuscript.

## Additional information

**How to cite this article:** Shim, T. S. *et al*. Dynamic designing of microstructures by chemical gradient-mediated growth. *Nat. Commun*, 6:6584 doi: 10.1038/ncomms7584 (2015).

## Supplementary Material

Supplementary Figures, Discussion and References Supplementary Figures 1-9, Supplementary Discussion and Supplementary References

Supplementary Movie 1 Time-resolved distribution of the fraction of monomer consumed by propagation of the reaction (*φ*_p_) and contours for *φ*_p_ = 0.1 in the absence of an additional source of oxygen during photopolymerization.

Supplementary Movie 2 Rotational and translational motions of PEGDA microneedles containing iron oxide nanoparticles under magnetic field.
