# Supplementary material for: Dynamic designing of microstructures by chemical gradient-mediated growth
Source: Nat Commun. 2015 Mar 13;6:6584. doi: 10.1038/ncomms7584 (PMC4382680; doi:10.1038/ncomms7584)
Supplement: Supplementary Figures, Discussion and References — Supplementary Figures 1-9, Supplementary Discussion and Supplementary References [file ncomms7584-s1.pdf]

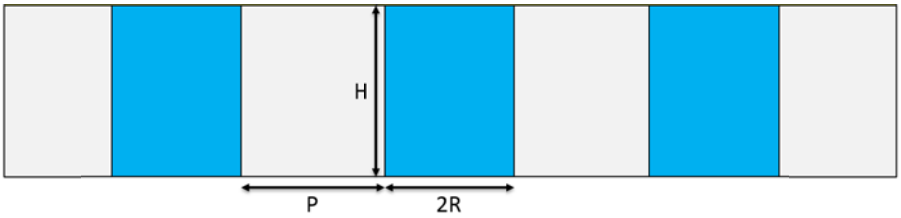

Supplementary Figure 1. Design for computations used to model photopolymerization with the reaction-diffusion process. The design is comprised of three reaction sites (blue) with widths of 2R and heights of H placed between idle (nonreactive) sites (gray) separated by P. The central reaction site is used to model experiments.

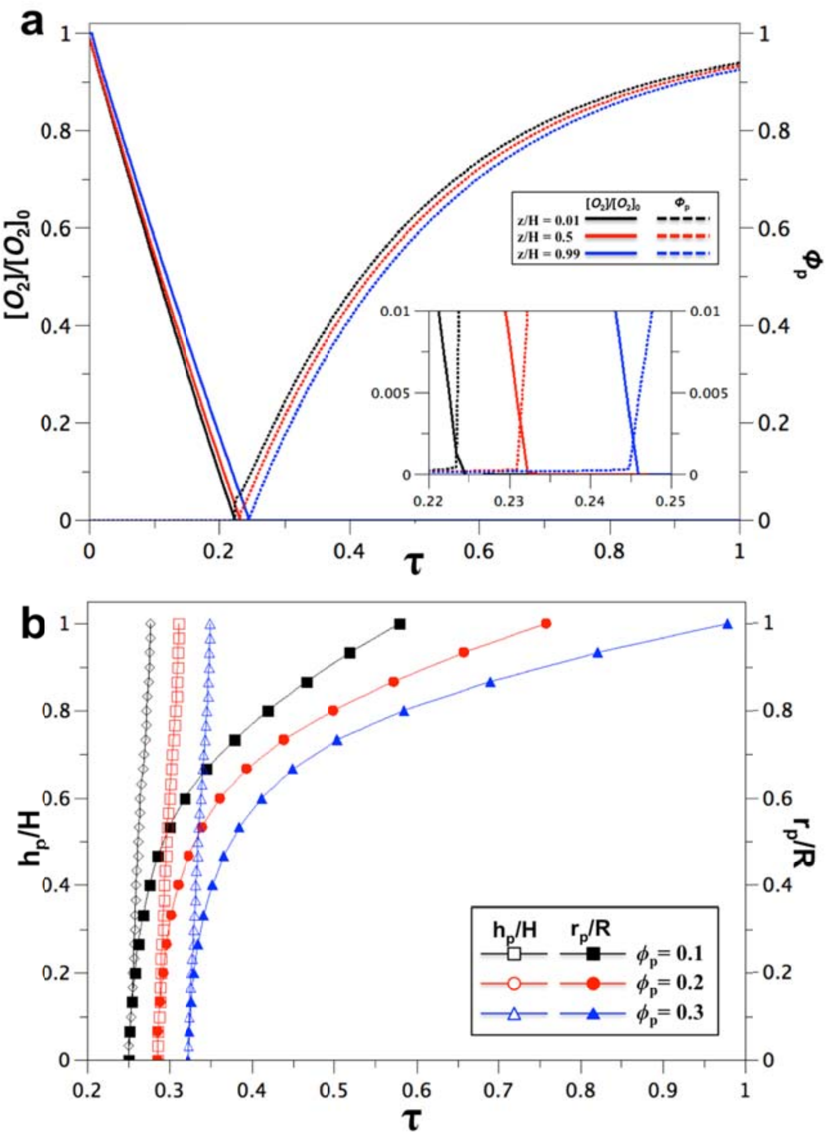

Supplementary Figure 2. Time-dependent concentrations of oxygen and monomer during polymerization. a, Normalized oxygen concentrations (solid lines) and fractions of monomer consumed by propagation of the reaction, ϕ_p = 1- [M]/$[M]_0$, (dotted lines) taken at three different positions of z/H (i.e., z/H = 0.01 (black), 0.5 (red), and 0.99 (blue)) and at r/R = 0. Propagation occurs as soon as oxygen is depleted. **b,** Normalized height and radius at which ϕ_p is 0.1, 0.2, and 0.3, respectively.

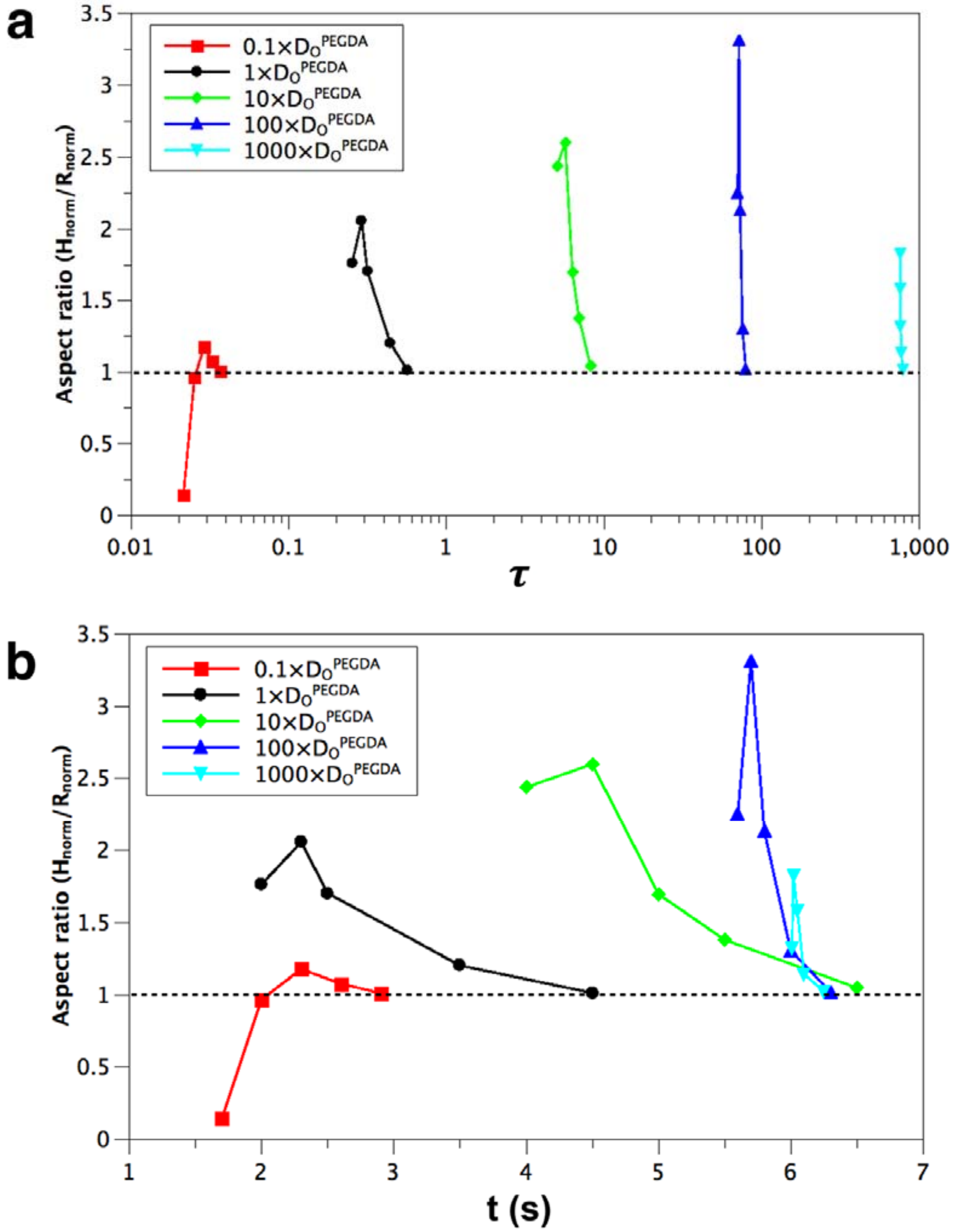

Supplementary Figure 3. Influence of D_O on growth. a, b Time-dependent change of the aspect ratio, $(h_p/H)/(r_p/R)$, of the contours with $\phi_p = 0.1$, for $D_O = 0.1\times D_O^{PEGDA}$ (red squares), $1\times D_O^{PEGDA}$ (black circles), $10\times D_O^{PEGDA}$ (green diamonds), $100\times D_O^{PEGDA}$ (blue triangles), and $1000\times D_O^{PEGDA}$ (cyan inverted triangles) where D_O^{PEGDA}, which indicates D_O of PEGDA, is 2.84×10^{-11} m^2s^{-1}. Abscissa is nondimensionalized time, τ (log scale) in (**a**) and dimensional time, *t*, (linear scale) in (**b**).

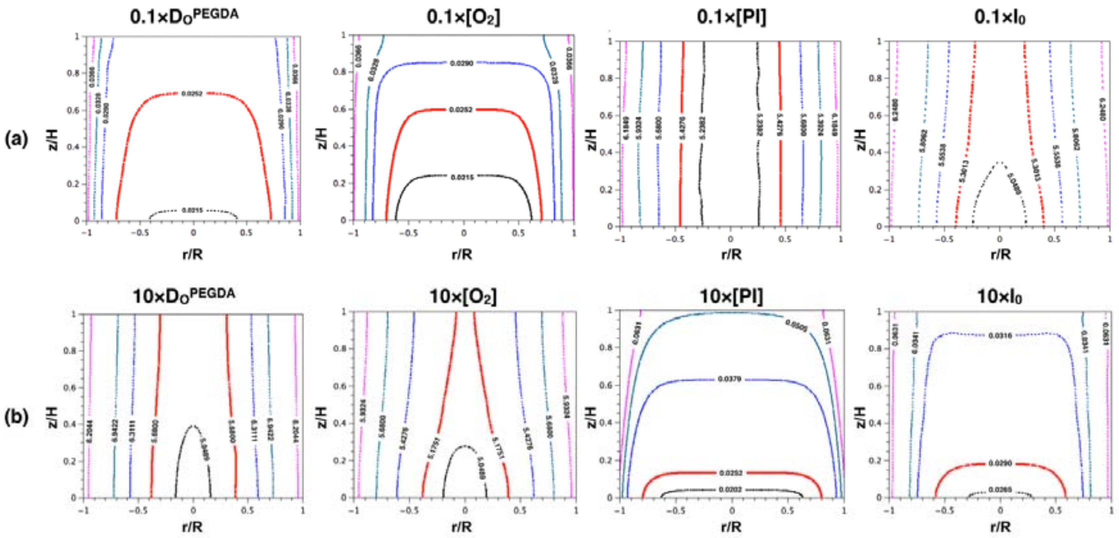

Supplementary Figure 4. Influence of various parameters on growth. a,b Time-dependent contours at which $\phi_p = 0.1$ at the reaction site. Abscissa and ordinate are normalized by the radius of the transparent window, R, and height of the monomer film, H, respectively. To investigate the influence of various parameters, D_O, [O_2], [PI], and I_0 are adjusted to have one order of magnitude smaller values in in **(a)** and one order of magnitude larger values in **(b)** than those in the experimental conditions: the values of D_O, [O_2], [PI], and I_0 in the experiment are 2.84×10^{-11}m^{2}s^{-1}, 1.5 mol m^{-3}, 1980 mol m^{-3}, and 4.73×10^{-4} mol m^{-2}s^{-1}, respectively.

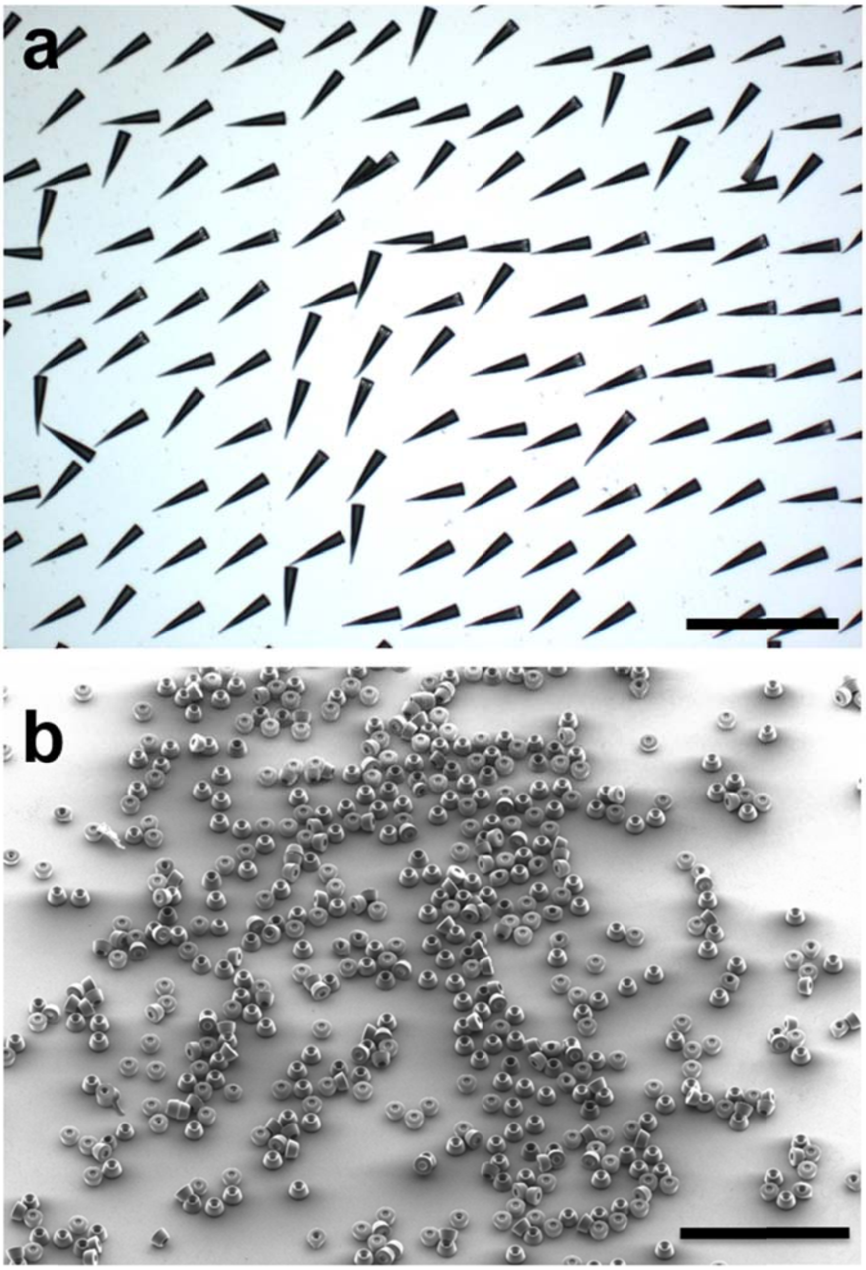

Supplementary Figure 5. Low-magnification image of microparticles created by bottom-center-to-top-edge growth pathway. a, Optical microscope image of triangular pyramid microparticles. **b,** SEM image of volcano-shaped microparticles. Scale bars indicate 500 μm.

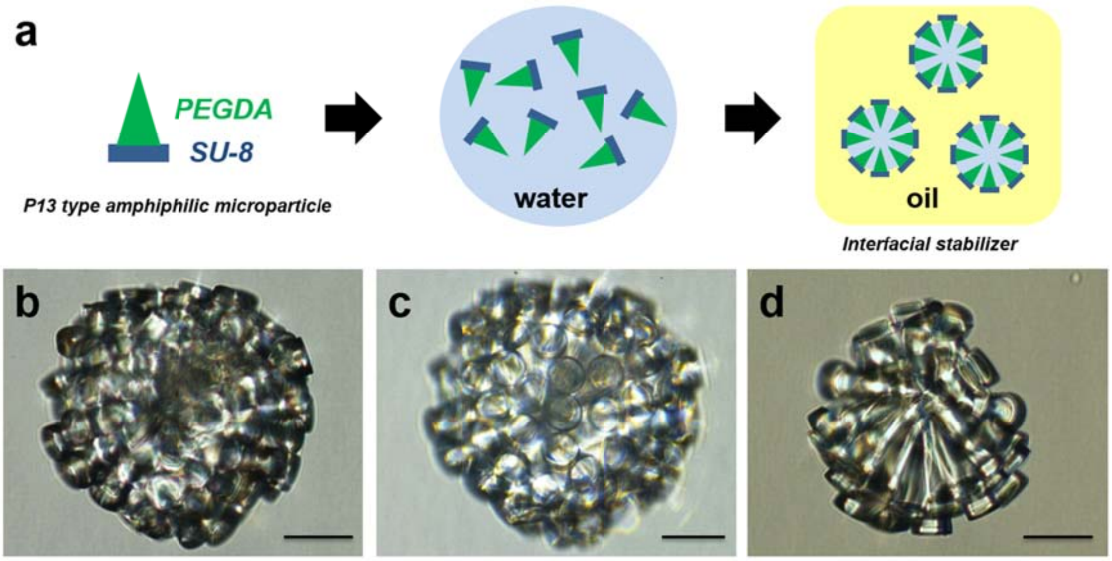

Supplementary Figure 6. Amphiphilic microparticles (P13-type). a, Schematics showing the stabilization of a water-in-oil emulsion interface by amphiphilic microparticles composed of a hydrophilic PEGDA cone and hydrophobic SU-8 disk. The microparticles were prepared by stepwise application of bottom-to-top (SU-8 disk) and bottom-center-to-top-edge (PEGDA cone) growths, which is referred to as P13-type. **b,c** Optical microscope images of an aqueous drop whose interface is stabilized with the microparticles. To prepare this, the aqueous suspension of microparticles was added to octanol, which was then emulsified by manual shaking. The images were taken in two different focal planes. **d,** Optical microscope image of aggregates of microparticles after water was completely diffused out. The microparticles maintain a micelle-like structure even after removal of water. All scale bars indicate 50 μm.

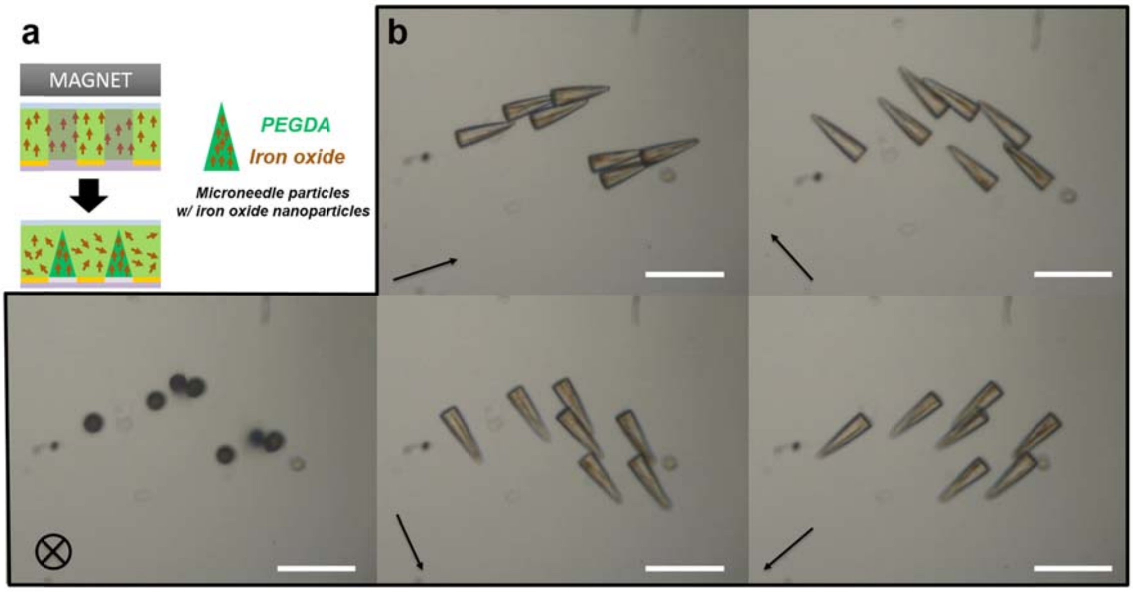

Supplementary Figure 7. Magnetoresponsive microneedles. a, Preparation of magnetoresponsive microneedles using bottom-center-to-top-edge growth. UV light was irradiated into a PEGDA suspension of weakly ferromagnetic iron oxide nanoparticles (brown arrow) under an external magnetic field, where the external field aligns the nanoparticles, thereby providing a permanent net magnetic moment to each microneedle after polymerization. **b,** Snapshots showing alignment of microneedles under the external magnetic field, where the field direction is denoted with an arrow in each image. All scale bars indicate 100 μm.

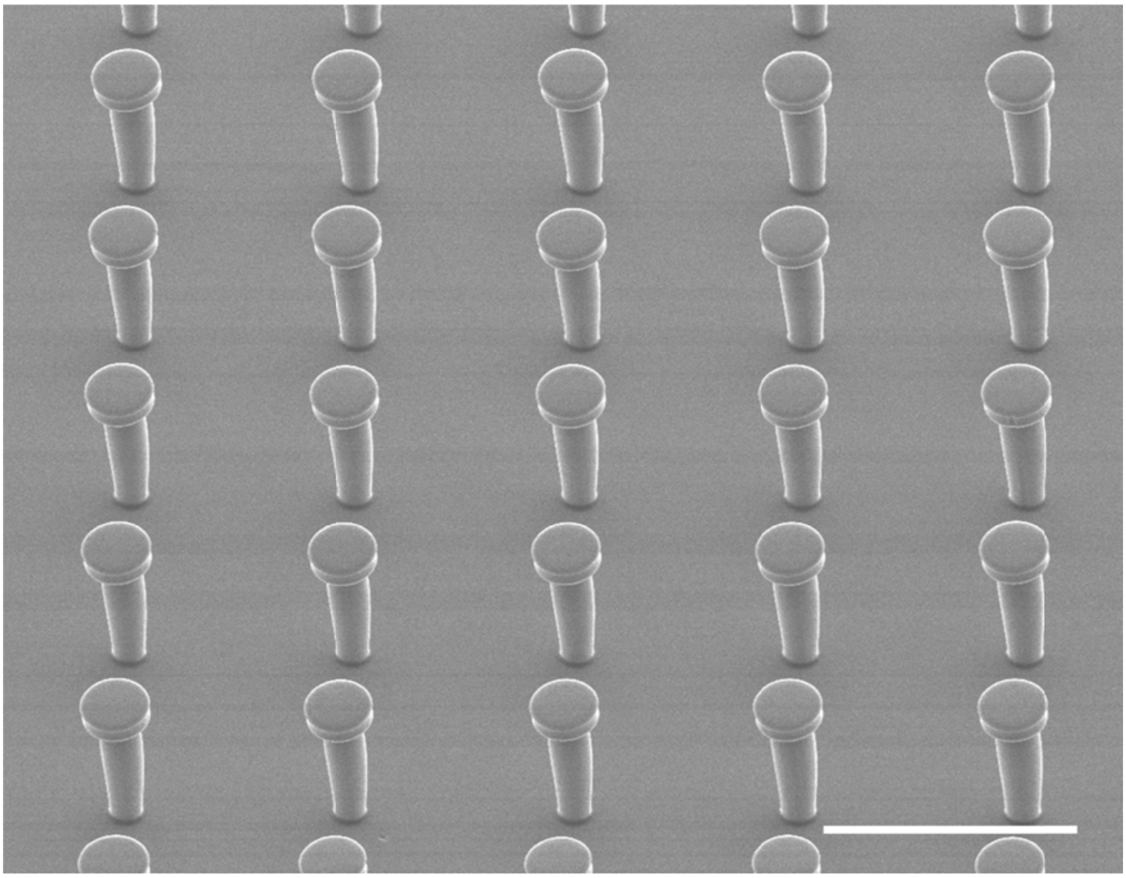

Supplementary Figure 8. Overhang microstructure arrays. SEM image of square array of overhanging structures composed of a top SU-8 disk and supporting ETPTA post. The disks are prepared by bottom-to-top growth and the posts are then prepared over the disks by center-to-side growth; therefore, the structures are P12-type. To turn over the structures and maintain their array, a thin film of polymerized ETPTA coated on a slide glass was used to cover the ETPTA monomers in the second growth, thereby showing strong adhesion between the posts and substrate. Scale bar indicates 100 μm.

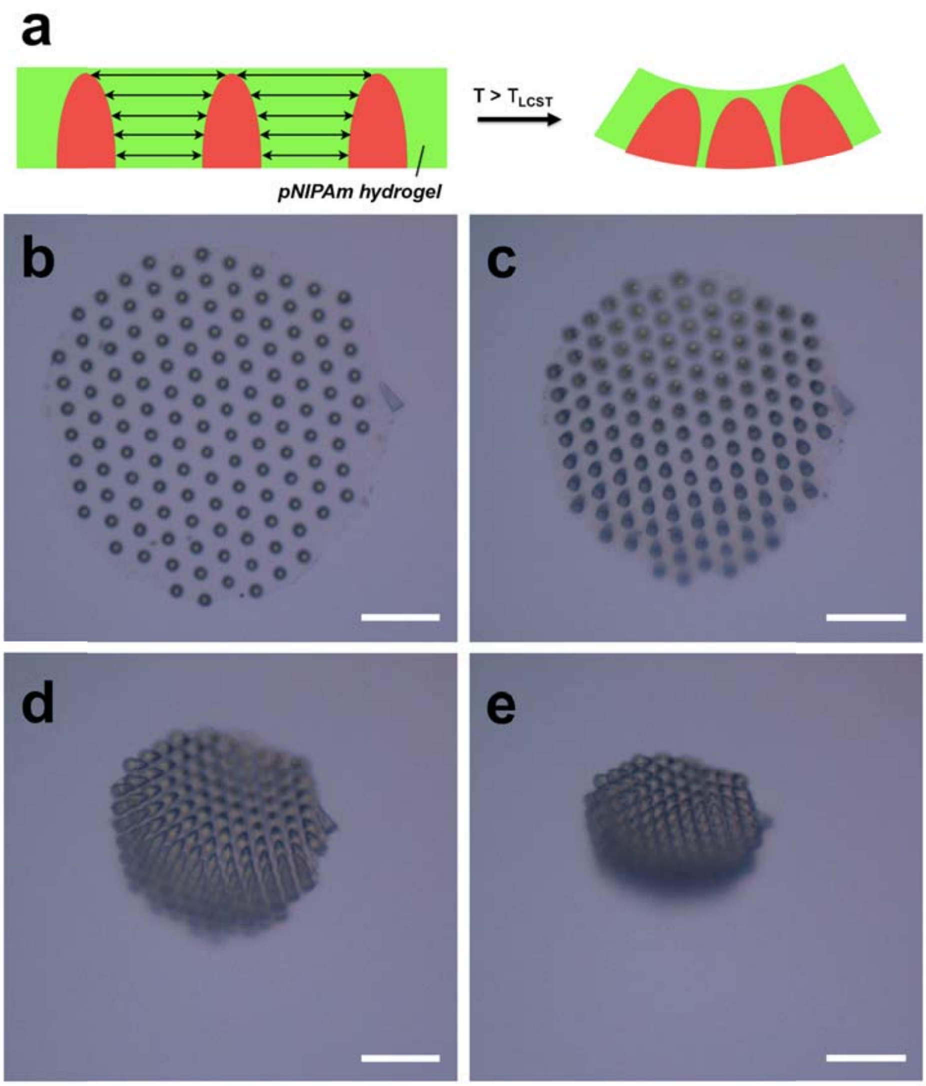

Supplementary Figure 9. Microcone-array-mediated folding of hydrogel film. a, Schematic illustration showing folding of microcone-embedded hydrogel film made of poly(N-isopropylacrylamide) (pNIPAAm). Above a lower critical solution temperature (LCST) of 32 °C, the pNIPAAm gel becomes shrunken, thereby leading to dense packing of rigid microcones. Therefore, the slope of the microcones determines the degree of curvature of the folded film. **b-e,** Series of OM images of the pNIPAAm film containing a hexagonal array of ETPTA microcones upon heating. All scale bars indicate 100 μm.

Supplementary Discussion

Modeling of Photopolymerization. Free-radical polymerization includes three reaction steps: 1) photolysis of the photoinitiator, 2) chain propagation, and 3) chain termination. The rate of photolysis, r_a, is proportional to the product of photoinitiator concentration, $[PI]$, and UV intensity, I, where I gradually decays along the propagation direction, z, since the photoinitiators absorb the light as described by the Beer-Lambert law:

$$r_a = \varphi\varepsilon[PI]I = \varphi\varepsilon[PI]I_0\exp(-\varepsilon[PI]z) \quad . \tag{1}$$

In this equation, φ is the quantum yield of radical production, ε is molar extinction coefficient of the photoinitiator at 365 nm, and I_0 is the initial intensity of light at 365 nm. The radicals, produced by photolysis, are eliminated by either bimolecular termination or oxygen termination. Therefore, when both reactions are second-order, the rate of chain termination, r_t, can be expressed as

$$r_t = k_t[\dot{R}]^2 + k_O[\dot{R}][O_2] \quad , \tag{2}$$

where k_t and k_O are the reaction constants for chain termination and oxygen inhibition, respectively, and $[\dot{R}]$ and $[O_2]$ are the radical and oxygen concentrations, respectively. Since the radical termination is much faster than propagation, we assume that $r_a = r_t$ from the quasi-steady-state approximation and obtain $[\dot{R}]$ as follows:[1]

$$[\dot{R}] = \frac{-k_O[O_2]+\sqrt{(k_O[O_2])^2+4r_ak_t}}{2k_t} \quad . \tag{3}$$

The rate of chain propagation is proportional to the product of the radical and monomer concentrations:

$$r_p = k_p[\dot{R}][M] \,, \qquad (4)$$

where [M] is monomer concentration. Therefore, as can be seen by combining Equations 3 and 4, the rate of chain propagation is governed by oxygen concentration. To calculate $[O_2]$ ($\mathbf{r}$, t), oxygen supplied by diffusion and consumed by termination reactions are considered:

$$\frac{\partial [O_2]}{\partial t} = \mathrm{D}_O \nabla^2 [O_2] - k_O [\mathrm{O}_2][\dot{R}] \,, \qquad (5)$$

where D_{O} is the diffusion constant of oxygen in monomers. We replace $[\dot{R}]$ with Equation (3) and solve this equation with COMSOL Multiphysics (COMSOL Inc.) to predict $[O_2]$ and $[\dot{R}]$, which are then used to calculate r_{p}, or the fraction of monomer consumed by propagation of the reaction, ϕ_{p}, from Equation (4). The design of the computational system is illustrated in Supplementary Figure 1; three reaction sites are included to reflect the influence of neighboring reaction sites.

Supplementary References

1. Dendukuri, D., Panda, P., Haghgooie, R., Kim, J. M., Hatton, A & Doyle, P. S. Modeling of Oxygen-Inhibited Free Radical Photopolymerization in a PDMS Microfluidic Device. Macromolecules 41, 8547–8556 (2008).
